# Nanoparticulate Tetrac Inhibits Growth and Vascularity of Glioblastoma Xenografts

**DOI:** 10.1007/s12672-017-0293-6

**Published:** 2017-04-10

**Authors:** Thangirala Sudha, Dhruba J. Bharali, Stewart Sell, Noureldien H. E. Darwish, Paul J. Davis, Shaker A. Mousa

**Affiliations:** 1 Pharmaceutical Research Institute, Albany College of Pharmacy and Health Sciences, 1 Discovery Drive, Rensselaer, NY 12144 USA; 2 Wadsworth Center, New York State Department of Health, Albany, NY USA; 3 Department of Clinical Pathology, Faculty of Medicine, Mansoura University, Mansoura, Egypt; 4 Department of Medicine, Albany Medical College, Albany, NY USA; 5 NanoPharmaceuticals LLC, Rensselaer, NY USA

## Abstract

Thyroid hormone as L-thyroxine (T_4_) stimulates proliferation of glioma cells in vitro and medical induction of hypothyroidism slows clinical growth of glioblastoma multiforme (GBM). The proliferative action of T_4_ on glioma cells is initiated nongenomically at a cell surface receptor for thyroid hormone on the extracellular domain of integrin αvβ3. Tetraiodothyroacetic acid (tetrac) is a thyroid hormone derivative that blocks T_4_ action at αvβ3 and has anticancer and anti-angiogenic activity. Tetrac has been covalently bonded via a linker to a nanoparticle (Nanotetrac, Nano-diamino-tetrac, NDAT) that increases the potency of tetrac and broadens the anticancer properties of the drug. In the present studies of human GBM xenografts in immunodeficient mice, NDAT administered daily for 10 days subcutaneously as 1 mg tetrac equivalent/kg reduced tumor xenograft weight at animal sacrifice by 50%, compared to untreated control lesions (*p* < 0.01). Histopathological analysis of tumors revealed a 95% loss of the vascularity of treated tumors compared to controls at 10 days (*p* < 0.001), without intratumoral hemorrhage. Up to 80% of tumor cells were necrotic in various microscopic fields (*p* < 0.001 vs. control tumors), an effect attributable to devascularization. There was substantial evidence of apoptosis in other fields (*p* < 0.001 vs. control tumors). Induction of apoptosis in cancer cells is a well-described quality of NDAT. In summary, systemic NDAT has been shown to be effective by multiple mechanisms in treatment of GBM xenografts.

**Electronic supplementary material:**

The online version of this article (doi:10.1007/s12672-017-0293-6) contains supplementary material, which is available to authorized users.

## Introduction

Glioblastoma multiforme (GBM) is an aggressive central nervous system tumor with high mortality [1, 2]. The tumor is also relatively chemoresistant [3, 4] and radioresistant [5]. Its invasiveness and asymmetry result in fewer than 30% of patients at presentation having tumors amenable to total resection [6]. The cancer is highly vascular, causing the evaluation of anti-angiogenesis in its management [7]. Against this background, it is understandable that a number of novel therapeutic approaches are under investigation [8–10].

Hercbergs and co-workers in a prospective, controlled clinical study developed evidence that GBM is thyroid hormone dependent, that is, medical induction of mild hypothyroidism slowed the course of the disease [11]. We reported that thyroid hormone, L-thyroxine (T_4_), was a proliferative factor in vitro in rat C6 and F98 cells and mouse GL261 cell line models for glioblastoma [12]. This effect was mediated by the cell surface thyroid hormone receptor we described on plasma membrane integrin αvβ3 [13, 14]. The deaminated T_4_ derivative, tetraiodothyroacetic acid (tetrac), blocked the proliferative action nongenomically initiated by T_4_ on these cells [12]. We also demonstrated that thyroid hormone was anti-apoptotic in glioma cells [15]. In studies of a human GBM cell line (U87MG), we defined discrete domains in the thyroid hormone receptor on αvβ3 that bound T_3_ and both T_4_ and T_3_ [16]. The T_3_ domain activated phosphatidylinositol 3-kinase (PI3-K), whereas mitogen-activated protein kinase (MAPK; ERK1/2) signal transduction pathway was activated by the T_4_/T_3_ domain. The latter supported U87MG cell proliferation.

We subsequently showed in a variety of xenografts that a formulation of tetrac covalently linked to a nanoparticle (Nanotetrac, Nano-diamino-tetrac, NDAT)—an agent thus restricted to the extracellular space and to action at the integrin—effectively slowed tumor growth and reduced vascularity [17–21]. The nanoparticle in this agent is biodegradable poly (lactic-co-glycolic acid) (PLGA). The antitumor effectiveness of NDAT involves pro-apoptotic activity and disruption of transcription of genes essential to a number of cancer cell survival pathways. The anti-angiogenic activity of NDAT involves disordering by several mechanisms of actions of endogenous growth factors, such as vascular endothelial growth factor (VEGF) [22], basic fibroblast growth factor (bFGF; FGF2) [22] and platelet-derived growth factor (PDGF) (S.A. Mousa, unpublished observations). NDAT also downregulates expression of the epidermal growth factor receptor (*EGFR*) gene [23]. Again, all of these actions are nongenomically initiated at integrin αvβ3.

In the present studies, we document the efficacy of systemic NDAT, acting via the thyroid hormone-tetrac receptor on integrin αvβ3 against human U87MG glioblastoma xenografts.

## Materials and Methods

### Cultured U87MG-luc Cells

Human glioblastoma U87MG-luc cells were a generous gift from MD Anderson Cancer Center, Houston, TX, and were grown in DMEM supplemented with 10% fetal bovine serum, 1% penicillin, and 1% streptomycin. Cells were cultured at 37 °C to sub-confluence and treated with 0.25% (*w*/*v*) trypsin/EDTA to effect cell release from culture flask. Cells were washed with culture medium, suspended in DMEM that was free of phenol red and fetal bovine serum, and counted. Medium and other culture materials were from Sigma-Aldrich (St. Louis, MO).

### Animals

Immunodeficient, female NCr nude homozygous mice aged 5–6 weeks and weighing between 18 and 20 g were purchased from Envigo (Indianapolis, IN). All animal studies were conducted at the animal facility of the Veteran Affairs Medical Center, Albany, NY, in accordance with and approved by institutional guidelines for humane animal treatment and according to the current guidelines. Mice were maintained under specific pathogen-free conditions and housed under controlled conditions of temperature (20–24 °C) and humidity (60–70%) and 12-h light/dark cycle with ad libitum access to water and food. Mice were allowed to acclimatize for 5 days prior to the start of study.

### Tetrac, NDAT

Tetrac was obtained from KareBay Biochem, Inc. (Monmouth Junction, NJ) and solubilized as previously described [19, 20], and NDAT was generated as previously described [19]. Both chemical structures are shown in the Supplemental Data. The nanoparticle diameter distribution, morphology, and zeta potential have also been reported [24]. The product consists of tetrac that is ether-bonded to a diaminoproprane linker via the outer ring hydroxyl group of tetrac. The linker is then attached by an amide bond to a nanoparticle of PLGA of approximately 150–200 nm. The agent is administered subcutaneously (s.c.) as a suspension in PBS. The amount of tetrac, itself, that is administered in NDAT is corrected for the contributions to molecular weight of the linker and PLGA nanoparticle.

### Treatment

In the first set of experiments, there were two discrete xenografts/animal and four animals in each of six experimental groups (control, void nanoparticle, tetrac (3 μg), tetrac (10 μg), NDAT (3 μg), NDAT (10 μg)). Cancer cells (2 × 10^6^), together with the respective control or treatment compound, were implanted s.c. in each flank of each animal. The control was PBS, and treatment compounds were void nanoparticle (volume = 10 μg NDAT dose), unmodified tetrac (3 or 10 μg), or NDAT (3 or 10 μg). NDAT at 10 μg/implant achieves an intratumoral tetrac equivalent concentration of approximately 10^−7^ M [25]. After humane sacrifice of animals at 16 days, tumors were harvested and hemoglobin concentration determined (see method below) to estimate graft vascularity. Tumor cell viability was estimated with In Vivo Imaging System (IVIS) scanning (see method, below).

In the second set of experiments, there were two grafts/animal and four animals for each experimental condition (control, NDAT). Xenografts consisted of 2 × 10^6^ cancer cells implanted s.c. in each flank of each mouse. After 2 days, the animals were treated daily with control (PBS) or NDAT (1 mg/kg) s.c. for 10 days. Animals were then humanely sacrificed and harvested tumors were subjected to histopathology examination.

### Estimation of Hemoglobin Content

Graft vascularity in the harvested tumors was estimated from an angiogenesis index of tumor cell lysate (tumor hemoglobin, mg/dL) measured with Drabkin’s reagent (Sigma-Aldrich) and with a hemoglobin standard, as described previously [21].

### Estimation of Tumor Cells’ Viability

Viability of cells in situ in harvested tumors from U87MG-luc cell-grafted animals was determined with luminescent signal measurement in an IVIS® apparatus (PerkinElmer Inc., Waltham, MA).

### Histopathology

Histopathology assessment of morphologic evidence of cell necrosis and apoptosis was done for slides prepared from the harvested tumors. Harvested xenografts were fixed in formalin, embedded in paraffin, and sectioned at the Wadsworth Center Core Histology Laboratory, New York State Department of Health. Paraffin-embedded tissue sections were stained with hematoxylin and eosin (H&E). Each tissue section was coded and examined by a board-certified pathologist who had no knowledge of the treatment exposure of the tissue (= blinded interpretation). The area of the tissue section was measured with a stage micrometer and the percentage of viable vs. necrotic tumor was estimated visually. The number of mitoses and apoptotic cells per high-power field was counted for five fields of viable tumor areas and averaged per tissue section. The degree of vascularization varied in the viable areas and this was graded numerically 1 to 4.

### Estimation of Tumor Volume and Weight

Tumor width and length were estimated with calipers at 3-day intervals and volume calculated from the standard formula (W × L^2^/2). Tumor weight was measured in harvested lesions following humane sacrifice of animals.

### Statistics

Statistical analysis was performed using one-way ANOVA, comparing the mean ± SEM from each experimental group with its relevant control group. Statistical differences at *p* < 0.05 were considered significant.

## Results

### Effect of Tetrac/NDAT Injected Intratumorally at Time of Implant on GBM Xenografts

#### Tumor Weight

**Fig. 1:**
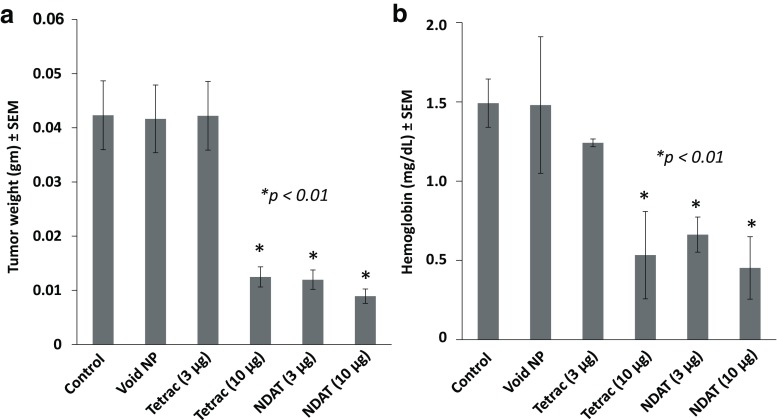
Effects at 16 days after single injection of U87MG cancer cells along with various treatments. There were four animals per group and two tumors per animal, *n* = 8. *Error bars* represent ±SEM and statistical significance is compared to control, *p < 0.01. **a** Tumor weights for control (PBS), void nanoparticle (NP), tetrac (3 μg), tetrac (10 μg), NDAT (3 μg), and NDAT (10 μg). **b** Hemoglobin content for the same groups

At animal sacrifice after day 16, harvested tumor weights were measured. Tetrac at 10 μg and NDAT at both 3 and 10 μg reduced tumor weight significantly compared to control tumors, *p < 0.01 (Fig. 1a).

#### Hemoglobin Assay

For a measurement of tumor vascularity, the hemoglobin content of cell extracts of xenografts revealed that NDAT treatment resulted in a 50% loss of tumor blood vessel content (Fig. 1b).

#### Cell Viability

**Fig. 2:**
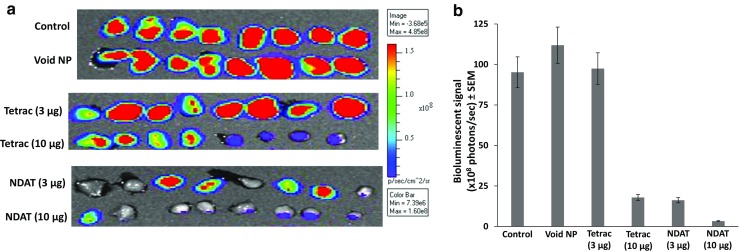
IVIS imaging of harvested tumors 16 days after single injections of U87MG-luc cancer cells along with various treatments; there were four animals per group and two tumors per animal. **a** Effects of control (PBS), void nanoparticle (NP), tetrac (3 μg), tetrac (10 μg), NDAT (3 μg), and NDAT (10 μg). The *vertical luminescence color bar* (*right side*) estimates viability, ranging from nonviable (*blue*) to fully viable (*red*). NDAT at 10 μg/implant approximates 10^−7^ M tissue tetrac equivalent drug concentration. **b** Quantitation of the tumor bioluminescent signal intensity in **a**. *Error bars* represent ±SEM compared to control, *n* = 8

IVIS scanning of excised U87MG-luc xenografts revealed virtually complete loss of viability of cells in the NDAT (10 μg) and substantial reduction in viability in grafts treated with 3 μg NDAT and 10 μg unmodified tetrac (Fig. 2a). Tetrac at 3 μg was ineffective. Figure 2b is the quantitative analysis of bioluminescence results depicted in Fig. 2a and shows an 80% reduction in cell viability with NDAT at 3 μg and with tetrac at 10 μg. NDAT at 10 μg completely eliminated tumor cell viability.

### Effect of Tetrac/NDAT Administered s.c. Daily on Established GBM Xenografts

#### Tumor Weight and Volume

**Fig. 3:**
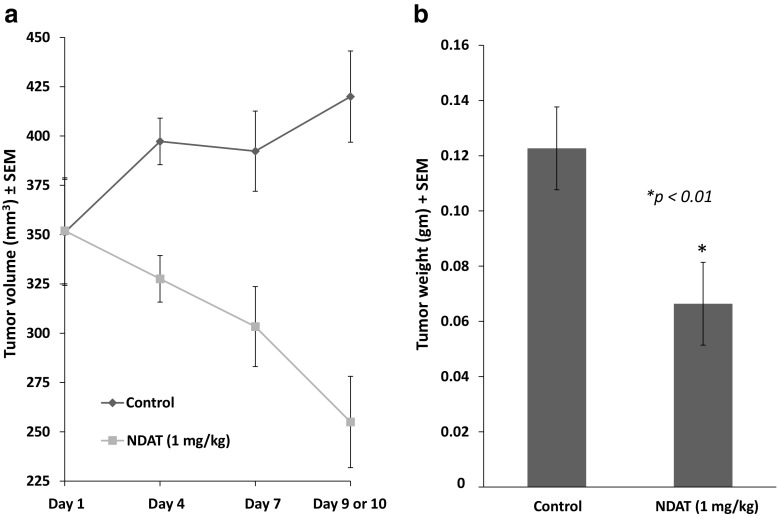
Effect of 10 days of daily s.c. NDAT (1 mg/kg) treatment on U87MG glioblastoma xenograft volume and weight. There were four animals per group and two tumors per animal, *n* = 8. *Error bars* represent ±SEM and statistical significance is compared to control. Three of the four control animals died on day 9, and their tumors’ volume and weight reflect 9 days of treatment. **a** Volumes of harvested tumors decreased by 60%, compared to controls. **b** There was a reduction in harvested tumor weight of 50% (*p < 0.01), compared to control lesions

At animal sacrifice after 10 days of treatment with NDAT, estimated volumes of harvested tumors decreased by 60%, compared to controls (Fig. 3a). In agreement with this measurement, there was a reduction in harvested tumor weight of 50% (*p < 0.01), compared to control tumors (Fig. 3b). We note that three of the four control animals died on day 9, and their tumors’ volume and weight reflect 9 days of treatment.

#### Histopathology

**Fig. 4:**
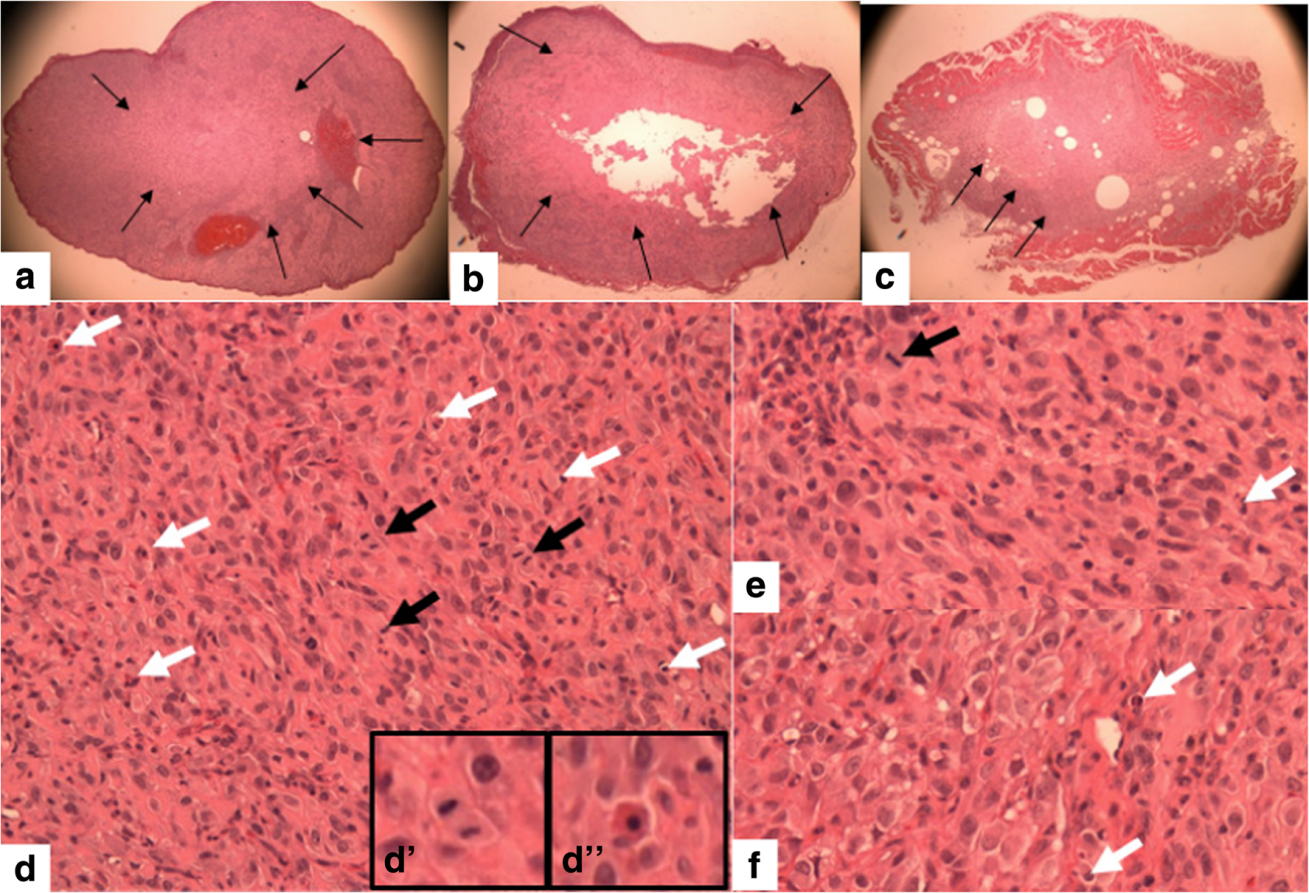
Histology showing effect of 10 days of daily s.c. NDAT treatment (1 mg/kg) on U87MG glioblastoma xenografts. **a**–**c**: Low power views of histologic sections of tumor xenografts harvested from control and NDAT-treated animals. **a** Sections from untreated animal, showing decreased cellularity in central area of tumor (*small arrows*), but not complete necrosis. **b**, **c** Sections from tumors of treated animals reveal extensive central necrosis involving 50% of tumor mass (**b**) or a smaller tumor (**c**) containing less than 30% viable cells. *Small arrows* show border of viable cells, and in **b**, part of the central necrotic area has been lost during processing of the tissue. *Lower 3 panels* (**d**–**f**) are high-power (400×) views of sections from tumors of NDAT-treated animals, showing cells undergoing necrosis (*black arrows*) and apoptosis (*white arrows*). *Insets D′ *and *D″* show mitosis and apoptosis, respectively. Apoptotic cells have pyknotic nuclei and condensed eosinophilic cytoplasm and show separation from adjacent viable cells. In necrotic areas, there is no cellular morphology or nuclear staining

**Fig. 5:**
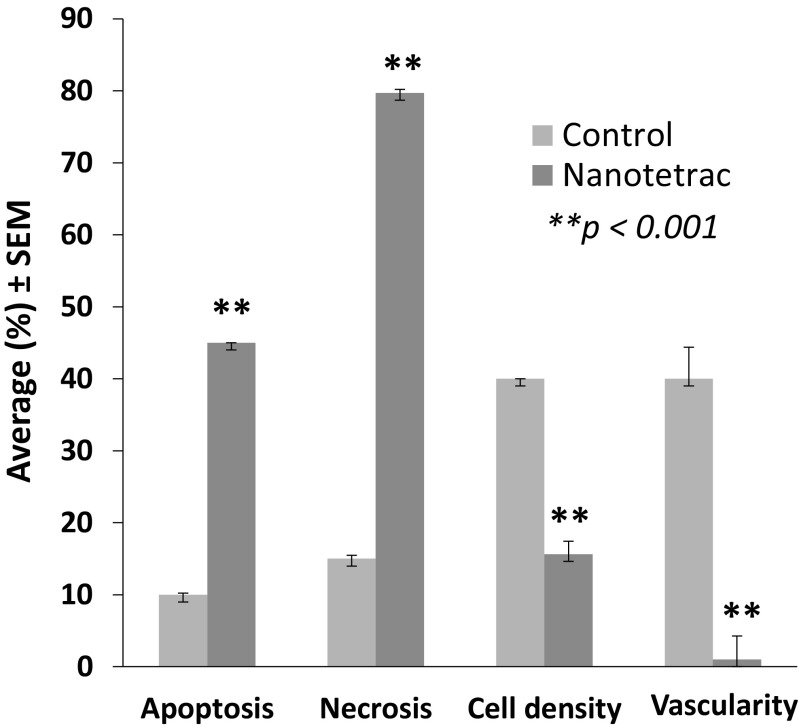
Induction of necrosis by 10 days of daily s.c. NDAT treatment (1 mg/kg) in U87MG glioblastoma xenografts significantly increased necrosis and apoptosis in various fields. As a result, cell density was reduced. The vasculature essentially disappeared from xenografts of NDAT-treated animals. Data were collected from four animals per group and two tumors per animal, *n* = 8. *Error bars* represent ±SEM and statistical significance is compared to control, **p < 0.001

**Fig. 6:**
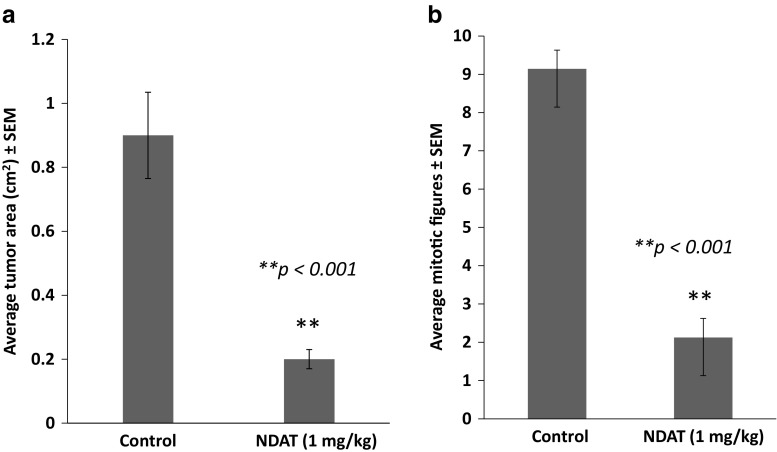
Effect of 10 days of daily s.c. NDAT (1 mg/kg) treatment on U87MG glioblastoma xenografts. Data were collected from four animals per group and two tumors per animal, *n* = 8. *Error bars* represent ±SEM and statistical significance is compared to control, **p < 0.001. Quantitative representation of **a** histologic estimates of tumor area and **b** average number of mitotic figures/field examined

Figure 4a–c shows necrosis at low magnification of control (a) and NDAT-treated tumors (b–c). There is extensive necrosis on histologic evaluations of U87MG tumors harvested at 10 days from NDAT-treated animals. That is, in tumors from NDAT-treated mice, there was loss of tissue/cell density and of nuclear structure, as well as dissolution of plasma membrane. Shown quantitatively in Fig. 5, such necrotic changes were present in many slide fields in up to 80% of tumor cells from treated animals (**p < 0.001 vs. control tumors). Estimated cell density in tumors exposed to NDAT was reduced by 60%, compared to controls (**p < 0.001). Percentages reported in these histologic interpretations are means of observations made on multiple fields of four slides of each xenograft. Figure 5 also indicates that regions were frequently identified in NDAT-treated tumors in which apoptotic changes were prominent in cancer cells. In such regions, 40% of cells were apoptotic (**p < 0.001 vs. untreated tumors). Such evidence includes cell shrinkage, patchy condensation of nuclear chromatin, and increased density of cytoplasm. Figure 5 indicates that essentially all of the vascularity of NDAT-exposed tumors had disappeared (**p < 0.001 vs. control tumors). Figure 6a shows that tumor areas in histologic cross-sections of excised whole xenografts were reduced by 80% in animals receiving NDAT daily × 10 days, compared to control (**p < 0.001). The number of mitotic cells/microscopic field was decreased by 77% in xenografts of NDAT-treated animals compared to control (**p < 0.001).

There were no examples of intratumoral hemorrhage in xenografts from drug-treated or control animals, and animal weights over the 10-day treatment period were unaffected by NDAT administration (data not shown).

## Discussion

A variety of chemotherapeutic [26–28] and anti-angiogenic [29] approaches have been used to manage GBM clinically. Success has been limited and a number of new chemotherapeutic and immunotherapeutic strategies are under development [30–34]. Further, the introduction of treatment agents directly by a catheter into tumors [35–38] offers the prospect of achieving high local concentrations of drug, bypass of the blood-brain barrier—although the latter may be porous about tumors [39, 40]—and limitation of systemic drug toxicity. Because of inherent radioresistance of this tumor, agents that radiosensitize are also of particular interest in management of glioblastoma.

In the present study, we determined the effectiveness of systemic NDAT (Nanotetrac, Nano-diamino-tetrac) against glioblastoma cell xenografts. The agent reduced tumor size promptly, effectively devascularized the tumors, and induced necrosis—presumably reflecting the loss of graft vascular supply—and apoptosis in cells not undergoing necrosis. Consistent with these findings, cell density in tumors from treated animals was reduced. In contrast to actions of other anti-angiogenic drugs tested oncologically [41–43], NDAT did not induce hemorrhage in the tumors or hemorrhagic events elsewhere. The basis of this finding in NDAT-exposed animals is not clear, but we propose that the effects on angiogenesis regulated from integrin αvβ3 can include systematic takedown of vasculature and not only inhibition of new blood vessel formation.

Cell necrosis in the context of devascularized tumors was expected. That apoptosis that was also induced by NDAT was also anticipated, given the existing evidence that NDAT is pro-apoptotic [14, 23]. The agent appears to affect both the intrinsic and extrinsic pathways of apoptosis.

The reduction in mitotic figures in tumors harvested from NDAT-treated animals is primarily a function of loss of cell viability. However, the drug is known to downregulate from its target/receptor on αvβ3 the transcription of a number of genes important to regulation of cell division [23, 44, 45].

In earlier in vitro studies, we showed that three rodent glioma cell lines proliferated in response to thyroid hormone and that this proliferative effect was blocked by tetrac [12]. T_3_ and T_4_ were equipotent in these experiments, but free T_4_ concentrations in human subjects are 50-fold those of free T_3_, so that the responsiveness of GBM to thyroid hormone, dictated by the hormone receptor site on integrin αvβ3, is T_4_ based. NDAT inhibits binding of T_4_ and T_3_ to the integrin as one mechanism of antitumor action, but NDAT has a number of antitumor properties that are independent of agonist thyroid hormone, as was pointed out above. In addition to rodent glioma studies, we have also reported that a human GBM cell line (U87MG) proliferates when thyroid hormone is added to the medium in amounts that produce a directly measured physiologic level of free hormone [16]. This hormonal action is mediated by mitogen-activated protein kinase (MAPK; ERK1/2).

Beyond anti-proliferation at the level of the tumor cell, a second important facet of the properties of NDAT is that it is anti-angiogenic by multiple mechanisms [14]. This should be particularly relevant to GBM because of its extensive vascularity. NDAT inhibits actions of vascular endothelial growth factor (VEGF) [22, 46], basic fibroblast growth factor (bFGF) [47], platelet-derived growth factor (PDGF) (S.A. Mousa, unpublished observations), and epidermal growth factor (EGF) [48]. Depending on the growth factor, these effects can involve gene transcription, growth factor release, or inhibitory crosstalk between the thyroid hormone-tetrac receptor on integrin αvβ3 and the vascular growth factor receptors clustered with the integrin on or in the plasma membrane.

A third facet of NDAT actions is that the agent increases radiosensitivity of human glioblastoma cells in vitro [49]. This has not been examined in xenografts. The radiosensitization action of NDAT involves inhibition of repair of double-stranded DNA breaks in the tumor cell.

Finally, the PD-1 (programmed death-1)/PD-L1 (PD-ligand 1) immune checkpoint is affected by physiological concentrations of T_4_, in that the hormone stimulates expression in a variety of cell lines of the *PD-L1* gene [50]. NDAT blocks this effect. This checkpoint is a focus of interest in immunotherapy of GBM [34].

What is apparent from the present studies is that NDAT has a panel of important anticancer and anti-angiogenic actions on glioblastoma xenografts. These effects are nongenomically induced at a cell surface target expressed generously by cancer cells and dividing endothelial cells. The agent is not cytotoxic and limitation of its actions to tumor and blood vessel cells explains a favorable side effect profile in preclinical studies. That is, histologic examination of organs such as the brain, heart, liver, and kidney in rodents exposed to high-dose NDAT for weeks has shown no abnormalities (S.A. Mousa, S. Sell, unpublished observations). We would also point out that the use of anti-angiogenic agents in GBM clinical management increases the risk of intratumoral hemorrhage [51, 52]. Despite the multifactorial anti-angiogenic properties of Nanotetrac, no hemorrhages occurred in the xenografts of Nanotetrac-treated animals in the present study.

A limitation of the current work is that it was carried out on xenografts of a single, widely used human glioblastoma cell line. On the other hand, we have previously shown that rat C6 and F98 glioma cell lines and mouse GL261 glioma cells proliferate in response to T_4_ and that this hormonal action on these cell lines is blocked by tetrac [12]. Further, human glioblastoma clinically responds to withdrawal of thyroid hormone [11, 53]. These observations are consistent with the NDAT observations we report in the current paper. The current studies were conducted on subcutaneous xenografts. We have compared the uptake of Cy5-labeled Nanotetrac in short-term (hours) by orthotopic and subcutaneous xenografts of U87-luc cells and have confirmed that tumoral uptake across the blood-brain barrier and in subcutaneous lesions is comparable (T. Sudha, D.J. Bharali, S.A. Mousa, unpublished observations).

## Electronic supplementary material

ESM 1 (DOCX 44 kb).
