# Supplementary material for: Nanoparticulate Tetrac Inhibits Growth and Vascularity of Glioblastoma Xenografts
Source: Horm Cancer. 2017 Apr 10;8(3):157–65. doi: 10.1007/s12672-017-0293-6 (PMC5413536; doi:10.1007/s12672-017-0293-6)
Supplement: Supplementary file 1 — (DOCX 44 kb) [file 12672_2017_293_MOESM1_ESM.docx]

**Supplementary**

**NANOPARTICULATE TETRAC INHIBITS GROWTH AND VASCULARITY OF GLIOBLASTOMA XENOGRAFTS**

Thangirala Sudha, Dhruba J. Bharali, Stewart Sell, Noureldien H. E. Darwish,

Paul J. Davis, Shaker A. Mousa


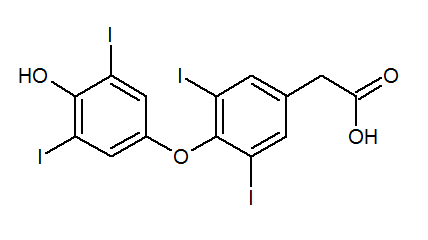


**Figure S1.** Chemical structure of tetrac (tetraiodothyroacetic acid).


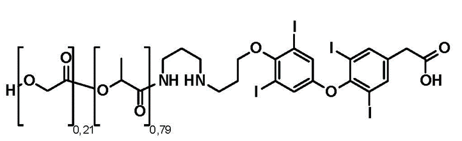


**Figure S2.** Chemical structure of NDAT (nano-diamino-tetrac). The subscripted numbers 0,21 and 0,79 mean that the lactic acid:glycolic acid ratio in the PLGA polymer averages 79:21.
